# Receptor-Like Protein Kinases Function Upstream of MAPKs in Regulating Plant Development

**DOI:** 10.3390/ijms21207638

**Published:** 2020-10-15

**Authors:** Zhe Wang, Xiaoping Gou

**Affiliations:** Ministry of Education Key Laboratory of Cell Activities and Stress Adaptations, School of Life Sciences, Lanzhou University, Lanzhou 730000, China; wangzh13@lzu.edu.cn

**Keywords:** MAPKs, RLKs, signal transduction, plant development

## Abstract

Mitogen-activated protein kinases (MAPKs) are a group of protein kinase broadly involved in various signal pathways in eukaryotes. In plants, MAPK cascades regulate growth, development, stress responses and immunity by perceiving signals from the upstream regulators and transmitting the phosphorylation signals to the downstream signaling components. To reveal the interactions between MAPK cascades and their upstream regulators is important for understanding the functional mechanisms of MAPKs in the life span of higher plants. Typical receptor-like protein kinases (RLKs) are plasma membrane-located to perceive endogenous or exogenous signal molecules in regulating plant growth, development and immunity. MAPK cascades bridge the extracellular signals and intracellular transcription factors in many RLK-mediated signaling pathways. This review focuses on the current findings that RLKs regulate plant development through MAPK cascades and discusses questions that are worth investigating in the near future.

## 1. Introduction

**Figure 1:**
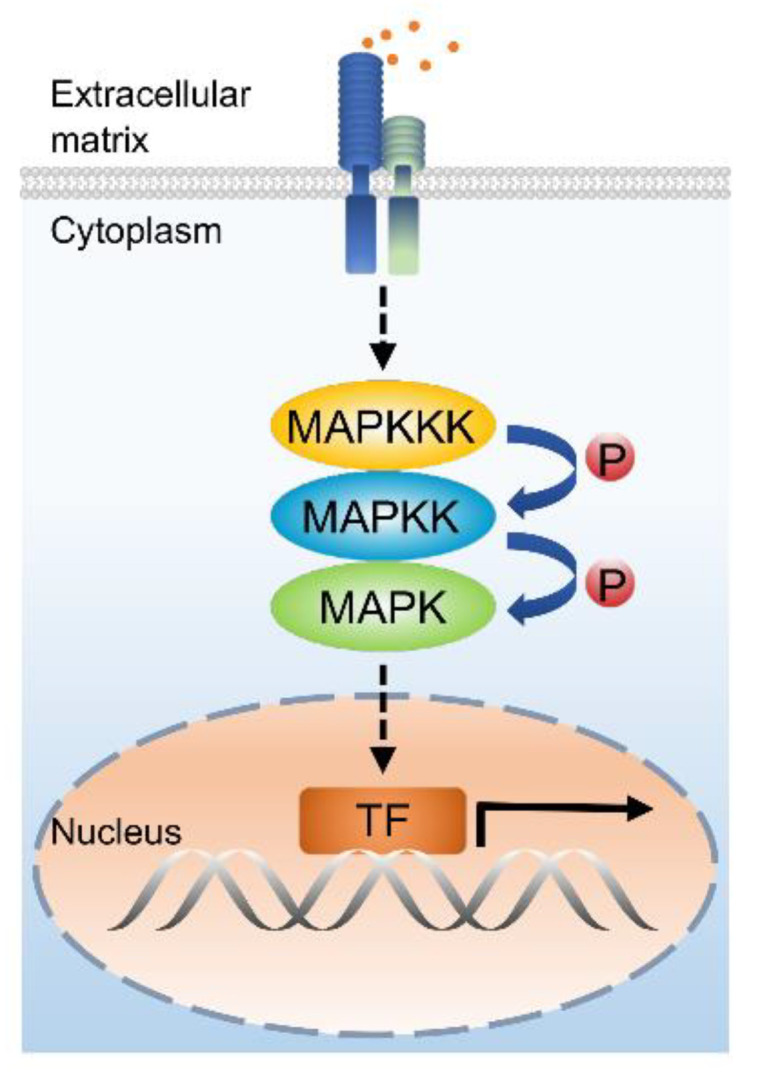
A general model of MAPK cascade functioning in plant development. A typical MAPK cascade contains three signaling components: a MAPK kinase kinase (MAPKKK), a MAPK kinase (MAPKK), and a MAPK. The MAPK cascade is activated by upstream regulators and transduces the signal to downstream transcription factors in the nucleus via sequential phosphorylation. The red dots represent extracellular signals that can be perceived by membrane-anchored receptors. The black arrow indicates gene expression. P, phosphorylation relay. TF, transcription factor.

In plants, gene expression is strictly regulated through complex networks of signaling pathways to modulate developmental processes and responses to environmental stresses. Mitogen-activated protein kinases (MAPKs) are a group of Ser/Thr protein kinases that were originally characterized as being involved in transmitting extracellular signals into the nucleus through signaling cascades [1]. Generally, a MAPK cascade consists of three kinases, a MAPK kinase kinase (MAPKKK), a MAPK kinase (MAPKK) activated by a MAPKKK, and a MAPK (hereafter named MPK) that is phosphorylated by a MAPKK [2] (Figure 1). MAPK cascades have been found in all eukaryotes [3]. For example, the grape (*Vitis vinifera*) genome possesses 88 MAPK cascade genes, including 7 MAPK kinase kinase kinases (MAPKKKKs), 62 MAPKKKs, 5 MAPKKs, and 14 MPKs [4]. The Arabidopsis (*Arabidopsis thaliana*) genome encodes 80 MAPKKKs, 10 MAPKKs, and 20 MPKs [5,6].

Plant MPKs are classified into four groups (A–D) according to the difference of the kinase structure and activation motif. The TxY motif of MPK is dually phosphorylated by dual-specificity MAPKKs. There are two kinds of TxY motif in the MPK subfamily. The MPKs with a TEY motif form three groups (A–C), and the TDY subtype is classified as a distinct group D [7]. Group A MPKs have been widely studied and found to be involved in environmental stresses, hormonal responses, and development in plants [8,9,10,11,12]. Group B MPKs are involved in microtubule organization, cytokinesis, and stress responses [13,14,15]. Tobacco (*Nicotiana tabacum*) NtMPK4, a group B MPK carrying an MEY instead of a TxY motif, is involved in ozone tolerance and the regulation of stomatal closure [16]. Group C MPKs have rarely been studied. It was reported that cotton (*Gossypium hirsutum*) MPK7 mediates defense responses against fungus and virus infection, and also promotes plant development possibly by response to phytohormone [17]. Instead of the TEY motif in groups A–C, group D MPKs have a TDY motif while lacking the C-terminal CD DOMAIN that is the docking site of MAPKKs [7]. Group D MPKs are involved in stress or defense responses [18]. Some findings suggest that group D MPKs mediate microtubule organization and stomatal movement [19,20,21].

Plant MAPKKs are also called MEKs or MKKs, which are divided into four groups (A–D) according to their amino acid sequence similarity [7]. Plant MAPKKs have a highly conserved phosphorylation site S/TxxxxxS/T that is S/TxxxS/T in human. Some group A MAPKKs regulate stress responses. For example, AtMEK1 mediates wounding-, drought-, and salt-induced signal transduction [22,23]. Inactive ZmMEK1 induces leaf senescence [24]. Group B MAPKKs have a conserved structural feature containing a nuclear transport factor 2 (NTF2) domain in the C-terminus region. AtMKK3 is a group B MAPKK, which is involved in stress response [25], immunity [26], and development [27]. AtMKK4/5 are the most thoroughly studied group C MAPKKs that are involved in many aspects of plant immunity [28,29], stress response [30], and development [31,32,33]. NaMEK2 regulates pollen germination and wound response in *Nicotiana attenuata* [34]. AtMKK7, a group D MAPKK, was reported to control plant development and stress response [35,36,37]. Plant MAPKKKs are classified into three groups (A–C) by homology of the kinase domain. In Arabidopsis, MAPKKKs form two main clades: MEKKs and RAF-like MAPKKKs [7]. MEKKs are typical MAPKKKs, such as YODA (YDA), MEKK1/4, and Arabidopsis NPK1-RELATED PROTEIN KINASE 1/2/3 (ANP1/2/3). These MEKKs are widely involved in development and response to environmental stresses [38,39,40,41,42,43]. Some studies have suggested that RAF-like MAPKKKs regulate defense and stress responses [28,44]. A recent study showed that RAF22 and RAF28 are necessary for the regulation of embryogenesis in Arabidopsis [45]. Sometimes, MAPK cascades have an upstream MAPKKKK, also a Ser/Thr protein kinase that may phosphorylate MAPKKKs. There are approximately 10 MAPKKKKs identified in Arabidopsis, some of which phosphorylate MAPKKs instead of MAPKKKs [46]. It was also found that some MAPKKKKs interact with MAPKKKs as scaffold proteins, and some of them are not involved in the MAPK cascades [46].

The first plant MAPK, MsERK1 that was identified in alfalfa (*Medicago sativa*), may be important in the mitogenic induction of symbiotic root nodules [47]. There has been an explosion of the research field of MAPKs in the past couple of decades, and many MAPK cascades and their functions were revealed in diverse plant species. MAPK cascades function in various processes of growth, development, immunity, and response to abiotic stress. For example, exogenous application of the elicitor peptide flagellin 22 (flg22) can activate the MEKK1-MPK4 cascade in Arabidopsis [48]. Two receptor-like protein kinases (RLKs), FLAGELLIN-SENSITIVE 2 (FLS2) and BRI1-ASSOCIATED RECEPTOR KINASE 1 (BAK1), form a receptor complex to perceive the flg22 signal and trigger the MAPK signaling cascade, thus regulating pathogen-associated molecular pattern (PAMP)-triggered immunity in Arabidopsis [49]. In addition, many reports showed that MAPK signaling regulates effector-triggered immunity (ETI) responses. An early study indicated that a fungus effector Avr9 can activate MAPKs in tobacco cells [50]. The MEK1/2-NTF6/WOUND-INDUCE PROTEIN KINASE (WIPK) cascade is involved in pathogen effector protein AvrPto-triggered disease resistance in tomato (*Solanum lycopersicum*) [51]. MAPK cascades are also involved in responses to abiotic stresses, such as cold, salt, and osmotic stresses. For instance, the MKK2-mediated signaling pathway is necessary for response to cold and salt stresses in Arabidopsis [52]. As one type of the most important signal transduction pathways, MAPK cascades participate in many aspects of plant growth and development. For example, in Arabidopsis, the MKK4/5-MPK3/6 cascade regulates embryogenesis, organ abscission, stomatal patterning, and lateral root emergence [41,53,54,55,56].

Consistent with their extensive functions, expression pattern analyses revealed that MAPKs are widely expressed in many plant tissues and organs, such as inflorescences, anthers, embryos, shoot and root apical meristems, root hairs and stomata [10,19,31,57,58]. Although most of them are ubiquitously expressed, some MAPKs are tissue- or organ-specific. For example, Arabidopsis *MPK6* is mainly expressed in inflorescences, stomata, pollen tubes, and root meristems [31,57,59,60]. It was a matter of controversy whether MAPKs are nucleus-localized because of their functions in transmitting extracellular signals into the nucleus. Many studies indicated that MAPKs are localized in the nucleus [61,62,63]. However, some reports showed that MAPKs entered the nucleus only when induced [64,65]. MAPKs were also found to be localized on the plasma membrane [66], suggesting that they may transduce signals mediated by some membrane proteins. Some MAPK modules are localized in the cytoplasm, including the cytoskeleton and the endomembrane system. For example, AtMPK18 interacts with ALPHA-GLUCAN PHOSPHORYLASE 1 (PHS1) which localizes in the cytoplasm to regulate microtubule organization [20]. Cytoskeletal protein MAP65-1 is a target of AtMPK4, which is also involved in microtubule organization [67].

Therefore, the MAPK pathways may integrate a variety of signals from different upstream regulators, including RLKs, phytohormones, cyclin-dependent kinases (CDKs), and other signal molecules. Some studies revealed the mechanisms of crosstalk between MAPK cascades and phytohormones, such as abscisic acid, auxin, brassinosteroids, jasmonic acid, salicylic acid, and ethylene [68,69,70,71]. There were also reports about other signaling pathways that can activate MAPK cascades. For example, calcium and nitric oxide signals can regulate plant physiological processes through the MAPK signaling pathways [72,73].

Receptor protein kinases (RPKs) are important mediators that regulate cell signaling pathways in metazoans [74]. This kind of proteins perceive upstream signals and transduce them to downstream signaling pathways, such as the MAPK cascades [75]. The first plant RLK was identified in maize (*Zea mays*) [76]. Then, RLKs were found in other plants, including Arabidopsis and *Brassica* [77,78]. More than 610 RLKs were identified in Arabidopsis after its genome was successfully sequenced at the end of 2000 [79]. A typical mature plant RLK harbors an extracellular domain perceiving extracellular signal, a transmembrane region to anchor it in the plasma membrane, and a kinase domain containing conserved amino acid residues present in Ser/Thr kinases [80]. RLKs in Arabidopsis were classified into at least 14 families according to their diverse extracellular domains [81]. The most common extracellular motif is leucine-rich repeat (LRR) that is thought to be involved in protein–protein interaction [79]. The LRR-RLK family contains at least 223 members in Arabidopsis, and was classified into 15 subfamilies according to the number of extracellular LRRs [82,83]. Typical RLKs are plasma membrane-localized signaling molecules that perceive extracellular signals to regulate plant growth, development, and response to environmental stimuli [80]. RLKs may function as upstream regulators and interact with MAPKs to transduce the perceived signals via sequential phosphorylation, regulating plant immunity and development [31,84,85,86] (Figure 1). In this review, we discuss how RLKs function as upstream regulators to control plant development through the MAPK cascades.

## 2. RLKs and MAPK Cascades Regulate Zygote Elongation, Asymmetric Division and Early Embryogenesis

**Figure 2:**
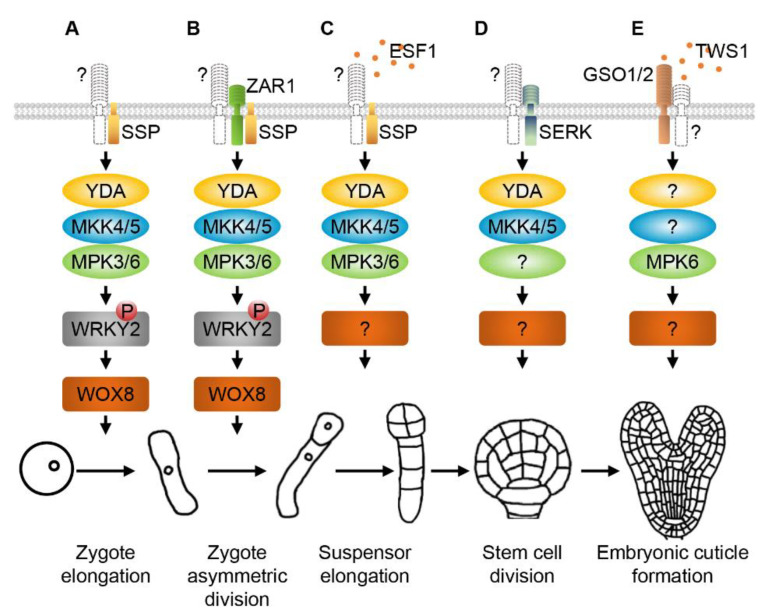
RLKs and MAPK cascades regulate embryo development. Membrane-associated receptor-like cytoplasmic kinase (RLCK) SHORT SUSPENSOR (SSP) regulates elongation of the zygote (**A**), asymmetric division of the zygote (**B**), and elongation of the suspensor (**C**) through the YDA-MKK4/5-MPK3/6 cascade. Leucine-rich repeat receptor-like protein kinase (LRR-RLK) ZYGOTIC ARREST 1 (ZAR1) is required to form a complex with SSP in controlling the asymmetric zygote division (**B**). The endosperm-secreted peptide EMBRYO-SURROUNDING FACTOR 1 (ESF1) regulates suspensor elongation together with SSP (**C**). A group of LRR-RLKs named SOMATIC EMBRYOGENESIS RECEPTOR-LIKE KINASES (SERKs) function upstream of YDA-MKK4/5 to control the division pattern of the ground tissue stem cells during early embryogenesis (**D**). The secreted peptide TWISTED SEED 1 (TWS1) is perceived by its receptor GASSHO1/2 (GSO1/2) to regulate embryonic cuticle formation through the YDA-MPK6 cascade (**E**). ? represents the unknown RLK, MAPK or transcription factor. P indicates the phosphorylation of WRKY2.

During early embryo development of Arabidopsis, the asymmetric division of the elongated zygote is an important event that determines the different developmental fate of the apical and basal cells. YODA (YDA), also known as MAPKKK4 in Arabidopsis, is a member of the MEKK subfamily. Loss-of-function of *YDA* leads to defective zygote elongation and apical-basal polarity of the embryo. The zygote of the *yda* mutant fails to elongate, resulting in two apical and basal cells with similar size. At the eight-cell stage, the *yda* embryo exhibits no obvious defects in the apical cell lineage but abnormal and short suspensor development. Oppositely, in plants with constitutively activated YDA (YDAac) the suspensor is longer than the wild type. Occasionally, the proembryo harboring YDAac shows irregular development or inhibited growth [87]. These data demonstrate that YDA-mediated signaling is essential for the asymmetric division of the zygote and early embryo development. Since the YDA-MKK4/5-MPK3/6 cascade was revealed in regulating stomatal development and patterning in Arabidopsis [31,88], it was reasonable to propose that MKK4/5 and MPK3/6 function as downstream signaling components of YDA during embryogenesis. Recently, it was shown that the *mkk4/5* double mutant exhibits strong defects in zygote elongation, asymmetric division of the zygote, and embryogenesis, similar to the *yda* mutant [33,55]. Similarly, the *mpk3/6* double mutant also generates an abnormal embryo without a distinguishable suspensor, similar to that of the *yda* and *mkk4/5* mutants [31]. These findings support that the YDA-MKK4/5-MPK3/6 signaling cascade controls zygote elongation, asymmetric division of the zygote, and development of the suspensor and early embryo proper in Arabidopsis (Figure 2A–C).

The WUSCHEL-RELATED HOMEOBOX (WOX) family transcription factors were found to be involved in early embryo development. In Arabidopsis, *WOX2* and *WOX8/9* are co-expressed in the zygote but restricted to the apical and basal cell, respectively [89,90]. The *wox1/2/3/5* quadruple mutant loses the apical structures of the embryo; the *wox8/9* double mutant shows abnormal division of the suspensor and the proembryo, resulting in arrested embryo development [90]. WRKY2, a zinc-finger transcription factor, can directly activate the transcription of *WOX8* and regulate zygote polarization and basal cell division patterns during early embryogenesis [91]. A recent study revealed that the activated MPK3/6 interact with and directly phosphorylate WRKY2 to up-regulate *WOX8* transcription in the zygote, which finally regulates zygote elongation and asymmetric division [92]. These results indicate that the YDA-MKK4/5-MPK3/6 signaling cascade regulates asymmetric zygote division and early embryo development by upregulating WRKY2-dependent expression of *WOX8*.

Some reports showed that RLKs are important regulators genetically upstream of canonical MAPK signaling cascades in controlling embryogenesis in plants. The sperm-originated and membrane-associated receptor-like cytoplasmic kinase (RLCK) SHORT SUSPENSOR (SSP) was reported as an upstream regulator of YDA in controlling elongation and asymmetric division of the zygote [93]. The *ssp* mutant shows similar but weaker embryonic defects when compared to the *yda* mutant, and hyperactive YDA can restore the suspensor phenotype of the *ssp* mutant [93]. Recent studies revealed that SSP genetically and biochemically interacts with YDA, and paternal SSP signaling activates the YDA-MPK3/6 cascade [86,92]. Defective zygote elongation and asymmetric division similar to *ssp*, *yda* and *mpk3/6* were also observed in the *mkk4/5* double mutant [33,55], indicating that MKK4/5 are key components in the SSP-YDA signaling pathway. All these findings demonstrate that SSP functions upstream of the YDA-MKK4/5-MPK3/6 cascade to regulate zygote polarity, elongation and asymmetric division (Figure 2A,B).

ZYGOTIC ARREST1 (ZAR1), an LRR-RLK expressed in the central cell, egg cell, synergids, zygote and endosperm, is required for *WOX8* expression, and thus the asymmetric division of the zygote and the fate of the daughter cells [94]. The expression patterns of *WOX2* and *WOX8* are impaired in *zar1*. Consistently, no *WRKY2* expression was detected in a *zar1* mutant with strong phenotype [94]. In addition, The Arabidopsis Gβ protein AGB1 functions as a scaffold protein to interact with ZAR1 and components of the YDA-MKK4/5-MPK3/6 cascade [86]. Moreover, ZAR1 can directly interact with SSP. These results demonstrate that a receptor complex formed by RLKs ZAR1 and SSP regulates zygote asymmetric division through the YDA-MKK4/5-MPK3/6 signaling cascade [86,94] (Figure 2B). Since the *zar1* zygote does not show obvious elongation defects, it is possible that ZAR1 is not involved in zygote elongation.

EMBRYO-SURROUNDING FACTOR 1 (ESF1) peptide family contains three members, including ESF1.1 to 1.3, that are secreted small cysteine-rich peptides specifically expressed in the central cell and embryo-surrounding endosperm cells [95]. RNA interference (RNAi) lines down-regulating the expression of three *ESF1* members show variable embryo proper patterning defects and a shortened suspensor, suggesting that the endosperm-secreted ESF1 peptides possibly regulate early embryo development in a non-cell-autonomous manner. In addition, genetic analyses revealed that ESF1 peptides function upstream of SSP and YDA to regulate suspensor length. Furthermore, reciprocal cross assays between *esf1* and single fertilization mutants lacking endosperm found that maternally produced ESF1 peptides in the central cell and endosperm are essential for early embryogenesis [95]. Collectively, these findings suggest that the extraembryonic maternal factor ESF1 may be perceived by a yet unknown receptor in the zygote and early embryo to control suspensor development through the SSP-YDA-MKK4/5-MPK3/6 signaling cascade (Figure 2C).

## 3. SERKs and MAPKs Regulate Division Patterns of Embryonic Stem Cells

During embryo development, the vascular precursors and ground tissue stem cells undergo transverse and asymmetric divisions to produce daughter cells that renew the stem cells and form the vascular tissue, endodermal cells, and cortical cells. For a long time, little was known about the underlying molecular mechanisms that strictly regulate the division of the stem cells. Although SOMATIC EMBRYOGENESIS RECEPTOR-LIKE KINASES (SERKs), a subfamily of LRR II-RLKs with a short extracellular domain containing five LRRs, have been discovered to be coreceptors of various RLKs in regulating many aspects of plant growth, development, and immunity [49,54,96,97,98,99,100,101,102,103,104,105,106], no evidence supported that SERKs regulate stem cell fate during embryo development.

Recently, however, a study demonstrated that SERKs control early embryogenesis in Arabidopsis [55]. The *serk1/2/3* triple mutant generates an arrested embryo because of the defective first asymmetric division of the vascular precursors and the abnormal second asymmetric division of the ground tissue stem cells. Interestingly, both the constitutively activated YDA and MKK5 can partially suppress the division defects of the ground tissue stem cells in the *serk1/2/3* embryo [55]. This study uncovered that SERKs and YDA-MKK4/5 function in the same signaling pathway to control cell fate of the ground tissue stem cells during early embryogenesis (Figure 2D). As mentioned above, MPK3/6 function downstream of YDA and MKK4/5 to regulate zygote elongation, the asymmetric division of the zygote, and suspensor development during early embryogenesis [33,86,92]. However, whether MPK3/6 are involved in regulating stem cell division and fate determination in embryogenesis still needs further investigation. On the other hand, the proposed ligand and receptor involved in this process have not been identified yet.

## 4. GSO1/2 and MAPKs Modulate Embryonic Cuticle Formation

The cuticle formed during embryogenesis surrounds the embryo and plays a critical role in preventing the embryo from fusing with the endosperm, which provides a relatively independent environment for proper embryo development. In the past decade, several key genes were identified in modulating embryonic cuticle formation. Loss-of-function of GASSHO1 (GSO1) and GSO2, two homologous LRR-RLKs with a long extracellular domain, results in seeds more twisted than the wild type. Detailed phenotypic analyses revealed that the cotyledons of the *gso1/2* double mutant adhere to each other and the endosperm tissue around the embryo at the late torpedo stage, and the mature embryo finally bends in reverse. Staining analysis showed that epidermis permeability of the cotyledons is impaired in the *gso1/2* double mutant because of defective cuticle formation during embryo development, which is at least partly responsible for the observed organ adhesion [107].

Recently, TWISTED SEED 1 (TWS1) was found to participate in the GSO1/2 signaling pathway as a non–cell-autonomous peptide hormone to modulate embryonic cuticle deposition on epidermal cells [108]. TWS1 is an embryo-expressed small peptide containing 81 amino acid residues. The *tws1* mutant produces twisted seeds and abnormal cotyledons adhered to the endosperm, similar to the *gso1/2* double mutant [109]. Embryo-expressed TWS1 precursors move into the endosperm where they are modified by ABNORMAL LEAF-SHAPE 1 (ALE1), a subtilisin-like serine protease whose expression is controlled by the transcription factor ZOUPI (ZOU), to generate mature TWS1 peptides [108,110,111]. Then, the mature TWS1 peptides are perceived by the receptors GSO1/2 in epidermal cells of the embryo. The TWS1-GSO1/2-mediated dialogue between the embryo and endosperm is essential for proper cuticle deposition on the embryo [108].

It was reported that the *mpk6* mutant also exhibits seed defects [112]. In addition, the phosphorylation level of MPK6 in *gso1/2* seeds is significantly reduced compared to the wild type [113]. Moreover, the *gso1/2 mpk6* triple mutant produces concave seeds and defective cotyledons with altered permeability, which is similar to the *gso1/2* double mutant. The impaired cotyledon permeability in the *ale1 mpk6* double mutant is not enhanced when compared with *ale1-4* [113]. The current findings support that MPK6, GSO1/2, and ALE1 may function in a common pathway, and MPK6 may act downstream of the GSO1/2-mediated signaling pathway [113]. The embryo of the *yda* and *mkk4/5* mutants also bends in reverse when compared with the wild type [55,87], suggesting that the YDA-MKK4/5-MPK6 cascade may also function downstream of TWS1-GSO1/2 to modulate cuticle deposition on the embryo, although further studies are still required in the future (Figure 2E). GSO1/2 also function as the receptor of two small peptides, CASPARIAN STRIP INTEGRITY FACTOR 1 (CIF1) and CIF2, to regulate the formation of the Casparian strip diffusion barrier [114,115]. A recent study suggested that SERKs may be involved in the CIF1/2-GSO1/2 signaling pathway as a coreceptor [116]. Whether SERKs function together with GSO1/2 to transduce the TWS1 signal in modulating embryonic cuticle deposition is worth further investigating in the near future.

## 5. ER family RLKs and MAPKs Regulate Anther Development

**Figure 3:**
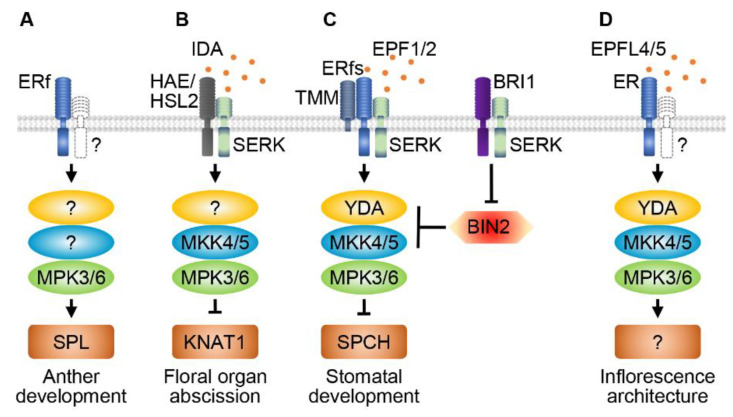
RLKs and MAPK cascades regulate reproductive and stomatal development. ERECTA family (ERf) members and MPK3/6 modulate anther development via activating the transcription factor SPOROCYTELESS (SPL) (**A**). HAESA/HAESA-like 2 (HAE/HSL2) and coreceptor SERKs perceive the INFLORESCENCE DEFICIENT IN ABSCISSION (IDA) peptide signal to control floral organ abscission by activating MKK4/5 and MPK3/6 (**B**). ERf, TOO MANY MOUTH (TMM) and SERKs form a receptor complex to sense the EPIDERMAL PATTERNING FACTOR 1/2 (EPF1/2) peptide ligands to control stomatal development and pattern via the YDA-MKK4/5-MPK3/6 cascade. BRASSINOSTEROID-INSENSITIVE 2 (BIN2) functions downstream of BRASSINOSTEROID INSENSITIVE 1 (BRI1) to repress the activity of YDA and MKK4/5 in controlling stomatal development (**C**). ER perceives the EPF-LIKE 4/5 (EPFL4/5) peptide signals to regulate inflorescence architecture via the YDA-MKK4/5-MPK3/6 cascade (**D**). ? represents the unknown RLK, MAPK or transcription factor.

In Arabidopsis, the diploid archesporial cells are formed in the L2 layer of the anther primordia. They divide and develop to form four lobes of the anther. Absence or defects in the archesporial cells usually lead to failure of the anther lobes and thus the male gametophytes. SPOROCYTELESS/NOZZLE (SPL/NZZ) is a crucial regulator of the archesporial cells. The loss function mutant *spl/nzz* shows defects in initiation and differentiation of both the microsporocytes and megasporocytes [117,118]. Ectopically expressed *SPL/NZZ* can induce microsporocytes in petals of the *agamous* mutant [119]. Some studies revealed reduced male fertility in *mpk6* and the double *mpk3^/+^ mpk6* mutant. Anthers of the *mpk6* and *mpk3^/+^ mpk6* mutants are significantly smaller than those of the wild type, and the pollen grains of *mpk6* are associated with the anther more tightly than the wild type, which suggests that MPK3/6 are involved in anther development of Arabidopsis [8,57]. In vitro and in vivo assays revealed physical interactions between SPL and MPK3/6 recently. In addition, MPK3/6 can phosphorylate SPL in vitro, and the phosphorylation of SPL by MPK3/6 is crucial for its functions in Arabidopsis anther development [120]. These results also suggest that additional components of the MPK3/6 cascade may be involved in anther development (Figure 3A).

ERECTA-family (ERf) RLKs, including ER, ERECTA-LIKE 1 (ERL1), and ERL2, have been discovered as essential regulators to control anther development. The *er erl1/2* triple mutant has small and incompletely differentiated anthers in flowers at stage 9 [121]. Both the *er erl1/2* and *mpk3^/+^ mpk6* mutants show some similar anther defects. For example, both of them fail to form one or more of the four anther lobes. Collectively, ERf-RLKs and MPK3/6 may function in a common signaling pathway and play important roles in specifying the archesporial cell identity or maintaining functions of the archesporial cells to generate the progeny cells [8] (Figure 3A). Some other LRR-RLKs were also found to be involved in anther development. For instance, EXTRA SPOROGENOUS CELLS (EXS)/EXCESS MICROSPOROCYTES 1 (EMS1) and SERK1/2 form a receptor/coreceptor complex that perceives the TAPETUM DETERMINANT1 (TPD1) peptide signal to control the differentiation of the microsporocytes and tapetal cells [104,122,123,124,125,126]. BARELY ANY MERISTEM 1/2 (BAM1/2) regulate the differentiation of the L2-derived cell types during microsporogenesis [127]. RECEPTOR-LIKE PROTEIN KINASE 2 (RPK2) guarantees the specification of the middle layer during early anther development [128]. Recently, it was reported that CLAVATA3 INSENSITIVE KINASE 1/2/3 (CIK1/2/3) function as coreceptors of BAM1/2 and RPK2 to regulate early anther development [129]. However, it is not known whether these RLKs affect archesporial cell initiation and differentiation through MAPK signaling.

## 6. RLKs and MAPK Cascades Regulate Organ Abscission

There is a small proportion of cells forming the abscission zone (AZ) in plants, which is the boundary between an organ and the plant, necessary for abscission to take place when the organ is senescent [130]. A peptide ligand–receptor system was found to regulate floral organ abscission in Arabidopsis. The *haesa haesa-like 2* (*hae hsl2*) double mutant fails to abscise its floral organs, which is similar to the *inflorescence deficient in abscission* (*ida*) mutant [131,132,133]. Neither overexpression of *IDA* nor application of synthetic IDA peptide can rescue the abscission deficiency of *hae hsl2*, indicating that *HAE* and *HSL2* are genetically epistatic to *IDA*, and IDA and HAE/HSL2 may control floral organ abscission through the same signaling pathway in Arabidopsis [131]. Further structural and biochemical analyses revealed that HAE functions as the receptor to transduce the IDA signal [101].

The *mkk4/5 RNAi* mutant and a *mpk6* dominant-negative mutant in the *mpk3* background produce non-abscising flowers, whereas constitutively activated MKK4/5 rescue the abscission-defective phenotype of the *hae hsl2* and *ida* mutants, suggesting that the MKK4/5-MPK3/6 cascade functions downstream of IDA-HAE/HSL2 in regulating organ abscission [32] (Figure 3B). However, whether YDA is also involved in the MKK4/5-MPK3/6 signaling cascade to control floral organ abscission is not known. Once the abscission program is initiated, AGAMOUS-like 15 (AGL15), a putative transcriptional repressor, is differentially phosphorylated through floral development by the MKK4/5-MPK3/6 signaling, which increases the expression of *HAE*, thus controlling the organ abscission process via a positive feedback loop [134]. All the *ida*, *hae hsl2*, and *mkk4/5* RNAi mutants exhibit defective drought-induced leaf abscission, indicating that the IDA-HAE/HLS2-MKK4/5 signaling is also necessary for drought-induced leaf abscission in Arabidopsis [135].

Two other regulators, EVERSHED (EVR), an LRR-RLK also known as SUPPRESSOR OF BIR1 1 (SOBIR1), and NEVERSHED (NEV), an ADP ribosylation factor, also play important roles in regulating the time of floral organ abscission in Arabidopsis by restricting the AZ cell size. The AZ is extended and organs abscise prematurely in the double *nev evr* mutant [136]. However, genetic analyses uncovered that mutation in *EVR* cannot rescue the abscission defects of *ida* and *hae hsl2* [136], implying that IDA-HAE/HSL2 may function in a parallel pathway with EVR and NVR, or EVR and NVR may act upstream of the IDA-HAE signaling pathway to regulate floral organ shedding. KNOTTED-LIKE FROM ARABIDOPSIS THALIANA 2/6 (KNAT2/6), two KNOTTED-like homeobox transcription factors, are activators of floral organ separation and suppressed by KNAT1 that is inactivated by the IDA signaling pathway [137]. Collectively, all these findings provide the evidence that IDA-HAE/HSL1/2 regulates KNATs through the MKK4/5-MPK3/6 cascade to timely modulate organ abscission (Figure 3B).

Three triple *serk* mutants, *serk1-1 serk2-1 bak1-5*, *serk1-1 bak1-5 serk4-1*, and *serk1-1 serk2-1 bak1-4*, exhibit defective floral organ abscission, similar to the *ida* and *hae hsl2* mutants, while the triple mutants *serk1-1 serk2-1 serk4-1* and *serk2-1 bak1-5 serk4-1* have normal floral organ abscission, indicating that these SERKs function redundantly and differentially to regulate organ abscission, and *SERK1* and *BAK1* are more important in this biological process [54]. Overexpression of *IDA* in the *serk1-1 serk2-1 bak1-5* mutant cannot rescue the floral organ abscission defects. Biochemical assays showed that SERKs can directly interact with HAE/HSL2, which can be enhanced by application of the IDA peptide, and IDA, HAE/HSL2 and SERKs can form a complex. Moreover, BAK1 and HAE transphosphorylate each other in their cytosolic domains [101]. These results demonstrate that SERKs function as coreceptors of HAE/HSL2 in regulating floral organ abscission, which is also supported by structural results that HAE can bind the IDA peptide more effectively in the presence of SERK1 [54,101]. In addition, constitutively activated MKK5 can restore the floral organ abscission defects of *serk1-1 serk2-1 bak1-5*. The current knowledge supports that SERKs are co-receptors of HAE/HSL2 in perceiving IDA, and function upstream of the MKK4/5-MPK3/6 cascade to regulate floral organ abscission (Figure 3B).

Interestingly, the regulation of organ abscission by peptide hormones has been also found in other plants. A very recent study revealed that the tomato (*Solanum lycopersicum*) phytosulfokine (PSK) signal is involved in stress-induced flower drop [138]. PSK was first described in *Asparagus* as a unique plant peptide growth factor [139]. Further studies suggested that the PSK signaling participates in plant growth and immunity [140,141]. Overexpressed phytaspase 2 (SlPhyt2), a precursor-processing protease of SlPSK, leads to premature abscission of flowers in tomato, and flower abscission increases to 70% as compared to 50% in the wild type under drought stress [138]. However, IDA seems not to contribute to stress-induced flower abscission in tomato because the expression of five *IDA* precursor genes is very low in the abscission zone and does not respond to drought stress [138]. Different from the IDA-regulated drought-induced leaf abscission in Arabidopsis [135], this study provides an insight on peptide hormone signaling in regulating the abscission process in other plants, although the downstream signaling components of SlPSK are still unknown.

## 7. RLKs and MAPKs Regulate Stomatal Patterning and Development

In Arabidopsis, the stomatal lineage begins with meristemoid mother cells (MMCs) transformed from a subset of protodermal cells. An MMC undergoes an asymmetric division to produce a smaller triangular meristemoid and a larger stomatal-lineage ground cell (SLGC). The SLGC can become a lobed pavement cell or initiate another asymmetric division to produce a satellite meristemoid that is oriented away from a stomatal precursor. The meristemoid loses its ability to reiterate asymmetric division after a variable number of amplifying divisions, then the meristemoid differentiates into a guard mother cell (GMC). The GMC divides once symmetrically to generate two guard cells. The mature guard cells control the size of the stomatal opening [142,143,144,145].

Small secretory peptide EPIDERMAL PATTERNING FACTOR 1 (EPF1) expressed in stomatal precursor cells was identified to control stomatal patterning by regulating asymmetric cell division. Overexpression of *EPF1* results in decreased stomatal density, while clustering of stomata and increased stomatal density exist in the *epf1* mutant [146]. The secretory peptide EPF2 that is related to EPF1 is expressed in MMCs, meristemoids, and GMCs to negatively regulate stomatal development. Overexpression of *EPF2* results in dramatically decreased stomata [147,148]. STOMAGEN, a peptide also known as EPF-LIKE 9 (EPFL9), was found to regulate stomatal development in another way. Different from *EPF1/2*, *STOMAGEN* is expressed in inner tissues of immature leaves instead of the epidermis. The stomatal density is reduced in leaf epidermis of the *stomagen* mutant [149,150,151]. STOMAGEN can bind the receptors by competing with EPF2 to antagonize the signaling pathway [152]. The ERf RLKs have been identified as the receptors of EPF peptides in regulating stomatal development [152,153]. Stochastically distributed and excess stomatal clusters are formed in cotyledon epidermis of the *er erl1 erl2* triple mutant. No double mutants of *ERf* members exhibit a phenotype with excess stomata similar to the *er* triple mutant [154,155], indicating that ER, ERL1, and ERL2 play an essential and redundant role for stomatal patterning and development. TOO MANY MOUTH (TMM), an LRR type receptor-like protein, was reported to suppress stomatal initiation together with the protease STOMATAL DENSITY AND DISTRIBUTION 1 (SDD1). The mutations in *TMM* cause clustered stomata and disrupted one-cell spacing stomatal patterning [88,143,156,157,158,159].

The *serk1-1 serk2-1 bak1-4* triple mutant produces clustered stomata in cotyledon epidermis, which is similar to the *er erl1 erl2* triple mutant [99,154,155]. BAK1 is the most important SERK in regulating stomatal development because the *serk* double or triple mutants containing mutation in *BAK1* can cause stomatal defects similar to the *er* triple mutant. Moreover, SERKs interact with ERfs and TMM, and EPFs can induce the interaction between SERKs and ERfs. In addition, exogenous application of EPF1/2 peptides cannot alleviate the stomatal defects. These results suggest that SERKs function as coreceptors of ERfs to control stomatal patterning and development [99].

MAPKs play essential roles in regulating stomatal pattern. YDA activity inhibits protodermal cells entering the stomatal lineage. However, YDA activity is required to regulate the generation of GMCs by meristemoids. Null *yda* mutants produce excess stomata [88]. On the other hand, constitutively activated YDA can suppress the stomatal phenotype of *serk1/2 bak1*, *tmm* and *sdd1* [88,99]. The *mkk4/5* RNAi plants show dramatic defects in stomatal development and patterning, with extensive excess stomata and no pavement cells, which is similar to the *yda* mutant. Accordingly, constitutively activated MKK4/5 inhibit stomatal cell fate initiation in the wildtype background, and greatly suppress the clustered stomata phenotype in the *yda* mutant. Similar to *yda* and *mkk4/5*, the *mpk6 mpk3RNAi* mutants produce excess stomata [31]. These data demonstrate that the YDA-MKK4/5-MPK3/6 signaling cascade acts downstream of the ERf-SERK receptor complex and plays a critical role to coordinate cell fate specification during stomatal development and patterning (Figure 3C). Another study showed that constitutively activated MKK7/9 in the MMCs can inhibit guard cell initiation, which is similar to that of constitutively activated MKK4/5. However, constitutively activated MKK7/9 driven by a GMC-specific promoter results in excess stomata. These results suggest that MKK7/9 may play an essential role in regulating guard cell initiation [160]. It is still unknown whether MKK7/9 function downstream of the ERf-SERK receptor complex.

Some studies revealed that brassinosteroid signal is also involved in regulating stomata formation because clustered stomata are produced in the brassinosteroid biosynthetic mutant *constitutive photomorphogenic dwarf* (*cpd*)*,* the receptor mutant *bri1*, and the gain-of-function signaling mutant *bin2-1* [161], respectively. BIN2, a shaggy-like kinase and key negative regulator in the brassinosteroid signaling pathway, can phosphorylate MKK4 to inhibit the MPK6 activity [162]. Another study indicated that BIN2 functions as a negative regulator of the MAPK cascade by phosphorylating YDA to control stomatal pattern and development. Furthermore, physiological and genetic assays suggested that BIN2 acts downstream of BRI1, but upstream of YDA-MKK4/5-MPK3/6, to regulate stomatal development [53]. These results imply that the YDA-MKK4/5-MPK3/6 cascade coordinates the brassinosteroid and EPF peptide signals to control stomatal pattern and development (Figure 3C). Recently, a study revealed that MAPKKK3 and MAPKKK5 transduce immune signals to the downstream module MKK4/5-MPK3/6, and this pathway and the YDA-MKK4/5-MPK3/6 signaling can antagonize each other [163], suggesting that different MAPK cascades, even though they share some signaling components, can coordinate immune responses and development processes.

SPEECHLESS (SPCH) is a bHLH transcription factor with a MAPK target domain, which can be phosphorylated by MPK3/6 in vivo [164]. No stomata can be formed in the *spch* mutant, similar to the phenotype of transgenic plants with constitutively activated YDA and MKK4/5 [31,88,165]. However, overexpression of *SPCH* without the MAPK target domain can produce excess stomata in the *mpk6* and *erl1 erl2* mutants [164]. Thus, SPCH functions as a crucial transcription factor downstream of the ERf-YDA-MKK4/5-MPK3/6 signaling pathway to control stomatal pattern (Figure 3C). Interestingly, it seems that different transcription factors may be employed by the MAPK cascade to regulate stomatal patterns in different plants. For example, *Brachypodium distachyon* BdYDA1 was reported to promote stomatal spacing patterning in *Brachypodium*. However, instead of BdSPCH1 and BdSPCH2, *Brachypodium* INDUCER OF CBF EXPRESSION 1 (BdICE1) that is expressed in the stomatal lineage cells, is a more likely the target of BdYDA1 [42].

## 8. RLKs and MAPK Cascades Regulate Root Development

MAPK cascades are also involved in signaling during Arabidopsis root development. Expression pattern analysis showed that *MPK6* is highly expressed in the meristem and transition zone of Arabidopsis roots [58]. Three *mpk6* knock-out mutants exhibit no root or short-root phenotypes. Disordered cell files caused by ectopic cell divisions were observed in the roots of *mpk6* [58]. Intriguingly, another study found that mutation in *MPK6* produces three types of seed: bigger seeds, smaller raisin seeds, and burst seeds. The seedlings from smaller raisin seeds show a short-root phenotype. However, the seedlings from bigger seeds have a longer primary root than that of the wild type, accompanied by increased lateral root initiation, and more and longer root hairs [112]. All these findings suggest that MPK6 plays critical roles during primary and lateral root development. Disordered cell files also result in thickened and short roots in *yda* [60]. *Arabidopsis ANP1/2/3*, the homologous MAPKKK genes of tobacco *NPK1*, were found to regulate cell division and growth [166]. The *anp2/3* double mutant exhibits defects in the root, which is caused by impaired microtubule organization [67]. The null *mpk4* mutant also shows microtubule-related phenotypes, such as cytokinetic defects in the primary root, which is similar to *anp2/3* [67,167]. Therefore, ANP2/3 may function upstream of MPK4 in controlling microtubule organization that affects root development. ABA-INSENSITIVE PROTEIN KINASE 1 (AIK1), a MAPKKK also known as MKKK20, is necessary for controlling cell division and elongation during primary root development. Roots of the *aik1* mutant are insensitive to abscisic acid treatment [168]. Biochemical analyses provided evidence that MKKK20 can interact with MKK3 and MPK18. Moreover, MKKK20 can phosphorylate MKK3 and MPK18. Primary roots of the *mkkk20 mpk18* and *mkk3 mpk18* double mutants are shorter than single mutants under treatment of microtubule-disrupting drugs. All these data suggest that the MKKK20-MKK3-MPK18 cascade is required for microtubule function in the root [27].

Several RLKs were reported to regulate primary and lateral root development. For example, ARABIDOPSIS CRIKLY 4 (ACR4) perceives the CLE40 peptide to regulate root meristem differentiation via controlling the size and position of root stem cell niche [169]. FERONIA (FER) functions as the receptor of peptide Rapid Alkalinization Factor 1 (RALF1) to regulate root growth, which may be related to endocytosis of plasma membrane proteins [170,171]. MUSTACHES (MUS) and MUSTACHES-LIKE (MUL), two LRR-RLKs, modulate lateral root primordial development by controlling the biosynthesis and remodeling of the cell wall [172]. However, there is no evidence yet that these RLKs regulate root development through MAPK cascades.

**Figure 4:**
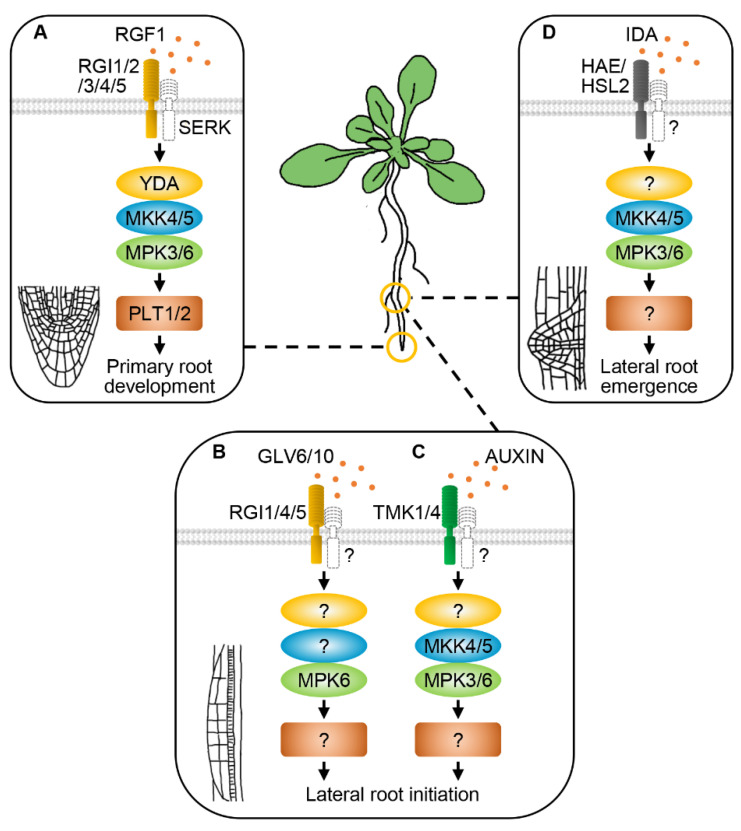
RLKs and MAPK cascades regulate root development. ROOT MERISTEM GROWTH FACTOR 1 (RGF1) INSENSITIVES (RGIs) and SERKs form a receptor complex to perceive the RGF1 peptide signal in regulating primary root development through the YDA-MKK4/5-MPK3/6 cascade (**A**). RGI1/4/5 and their ligands GOLVEN 6/10 (GLV6/10) control lateral root initiation by activating MPK6 (**B**). TRANSMEMBRANE KINASES 1/4 (TMK1/4) perceive auxin to regulate lateral root initiation through the MKK4/5-MPK6 cascade (**C**). The IDA-HAE/HSL2 ligand–receptor pairs act upstream of MKK4/5-MPK3/6 to regulate lateral root emergence (**D**). ? represents the unknown RLK, MAPK or transcription factor.

Recently, several studies revealed that MAPK signaling pathways also function downstream of RLKs to regulate primary root development. ROOT MERISTEM GROWTH FACTORS (RGFs) are a group of quiescent center-expressed Tyr-sulfated peptides that regulate primary root development via controlling root meristem size. Roots of the *rgf* mutants are extremely short, demonstrating that RGFs are crucial to postembryonic root development [173]. RGF1 INSENSITIVES/RGF RECEPTORS (RGIs/RGFRs) are a clade of LRR-RLKs containing five members, which function as receptors of RGF1 to regulate root meristem [100,102,174]. The primary root of the *rgi1/2/3/4/5* quintuple mutant is significantly shorter than the wild type because of the shrunken root meristem. Moreover, the *rgi* quintuple mutant is insensitive to RGF1 treatment. Upon perception of RGF1, phosphorylation and ubiquitination of RGI1 occur to transduce the signal. These findings demonstrate that RGIs sense the RGF1 signal, which is crucial for maintaining root meristem size and primary root development [100]. The stability of RGIs is strictly regulated by two ubiquitin-specific proteases, UBP12 and UBP13. UBP13 can directly interact with RGI1 and counteract the ubiquitination and degradation of RGI1 [175]. The *ubp12/13* double mutant is completely insensitive to RGF1 treatment, similar to the *rgi* quintuple mutant [175]. Genetic, biochemical and structural results revealed that RGIs recruit SERKs as coreceptors to perceive the RGF1 signal in regulating root meristem [100,102,174]. A very recent study revealed that constitutively activated YDA can rescue the phenotype of the *rgi1/2/3/4/5* mutant. The *mkk4/5* double mutants exhibit a smaller root meristem, a defect very similar to *rgi1/2/3/4*. Moreover, the *mkk4/5* mutants are partially insensitive to RGF1 treatment, suggesting that MKK4/5 may regulate root meristem size in the same signaling pathway with RGIs. Genetic and physiological analyses further implied that MPK3/6 control root meristem size downstream of MKK4/5 [176,177]. The expression of PLETHORA1/2 (PLT1/2), two critical transcription factors regulating root stem cell niche [178], is dramatically reduced in both of the *rgi* quintuple and *mkk4/5* double mutants [100,176]. These data suggest that the RGI-SERK receptor complex senses the RGF1 signal to regulate root meristem size and primary root development through the YDA-MKK4/5-MPK3/6 signaling cascade (Figure 4A).

RGF peptides are also required for lateral root initiation. GOLVEN 6 (GLV6), also named RGF8 and CLE-LIKE 2 (CLEL2), is expressed in early steps of the formation of lateral root primordium. Overexpression of *GLV6* results in decreased emergence of lateral roots. Treatment with the GLV6 peptide impairs the first asymmetric cell division during primordium formation [179]. Recently, GLV6/10 were found to participate in lateral root initiation, and RGI1/4/5 may function as receptors of GLV6/10 in controlling this process. Loss-of-function of *GLV6*/*10* increases the asymmetric cell division during lateral root initiation. Consistently, seedlings overexpressing *GLV6* lack visible lateral roots because of ectopic anticlinal division of pericycle cells. However, defective pericycle divisions caused by *GLV6* overexpression are suppressed in the *mpk6* mutant. Moreover, GLV6 can induce MPK6 phosphorylation and activation, which is dependent on the RGI receptors [180]. These results indicate that MPK6 is a downstream effector of the GLV6/10-RGI1/4/5 pathway during lateral root initiation (Figure 4B).

Extracellular auxin perception and signaling can be mediated by TRANSMEMBRANE KINASES (TMKs), a group of LRR-RLKs, to regulate apical hook formation in Arabidopsis, representing a new mechanism of auxin perception [181]. Exogenous auxin can induce lateral root initiation in the wild type, but is ineffective in the *tmk1*/*4* double mutant. The *mkk4/5* mutants are also insensitive to exogenous auxin regarding lateral root initiation. Biochemical assays showed that TMK1/4 can bind MKK4/5. Moreover, auxin can induce the phosphorylation of MKK4/5 and MPK3/6, which depends on TMK1/4. Taken together, these data indicate that auxin signaling regulates lateral root initiation also via the MKK4/5-MPK3/6 cascade [182] (Figure 4C).

Lateral roots can be properly formed only when the initiated primordia successfully develop and go through the surrounding cell layers. The *ida*, *hae,* and *hsl2* single mutants display reduced lateral root density, implying that HAE and HSL2 regulate lateral root development possibly through different pathways. IDA and HAE regulate the degradation of pectin, a component of cell wall. IDA, HAE, and HSL2 together regulate the transcription of cell wall remodeling genes. Collectively, the ligand–receptor complex IDA-HAE/HSL2 is necessary for lateral root emergence [183]. Recently, it was reported that the *mpk3/6* double mutant exhibits reduced lateral root density, a similar phenotype found in the *mkk4/5* mutant [56]. Detailed analyses revealed that the lateral root primordia fail to go through the endodermis, cortex and epidermis in *mpk3/6* and *mkk4/5*, which results in the reduced lateral root density. Meanwhile, constitutively activated MKK4/5 driven by a *HAE* or *HSL2* promoter can rescue the emergence defects of lateral roots in the *ida* and *hae hsl2* mutants [56]. In summary, the current results support that the IDA signal is perceived by HAE/HSL2 to regulate lateral root emergence through the MKK4/5-MPK3/6 signaling cascade (Figure 4D).

## 9. RLKs and MAPK Cascades Function in Other Plant Developmental Processes

The shoot apical meristem (SAM) contains a collection of undifferentiated cells, which produces primordia developing into all the above-ground organs of a plant. RLKs and MAPKs are also involved in maintaining the SAM. Overexpression of *MKK7* leads to collapsed and defective SAM. The process of de-etiolation induced by light is enhanced in the *mkk7* and *mpk6* mutants, suggesting that MKK7 and MPK6 function as negative regulators of meristem activity [36]. The CLAVATA-WUSCHEL (CLV-WUS) feedback loop is the classic regulatory pathway that maintains the homeostasis of the SAM [184,185]. The CLV3 peptide signal is transduced by RLKs, including CLV1 and RPK2, and CLV2/CORYNE (CRN) heterodimer consisting of the receptor-like protein CLV2 and the receptor-like cytoplasmic protein kinase CRN, to repress the expression of transcription factor WUS. CIKs, another group of LRR-RLK, function as coreceptors of CLV1, RPK2 and CLV2/CRN to maintain the SAM [186]. However, how the CLV3 signal is transmitted from the receptors on the plasma membrane to the nucleus-localized WUS is still largely unknown. It was reported that the CLV3 signaling can activate MPK6 to maintain SAM homeostasis [187]. A recent study showed that exogenous CLV3 can activate MPK3/6, and the *mpk3/6* double mutant is insensitive to CLV3 treatment [10]. The transcription levels of WUS-target genes, such as *ARABIDOPSIS RESPONSE REGULATOR 7/15* (*ARR7/15*), *GROWTH-REGULATING FACTOR 6* (*GRF6*) and *YABBY 3* (*YAB3*), are decreased in *mpk3/6*. Treatment with the CLV3 peptide can inhibit the SAM in the wild type. However, the SAM is still normal in most *mpk3/6* mutants treated with exogenous CLV3. Moreover, the size of the SAM in *mpk3/6* is larger than the wild type [10]. All these findings suggest that the CLV3-CLV1 signaling pathway regulates stem cell homeostasis in the SAM possibly via activating a MAPK cascade including MPK3/6.

The ERf members regulate several biological processes, including male and female reproductive organ development, stomatal development, and inflorescence architecture [121,144]. Loss-of-function mutation in *MPK3*/*6* and *MKK4/5* results in clustered inflorescence, which is similar to the *er* mutant. The *er-105 mpk6* double mutant exhibits enhanced inflorescence defects when compared with the *er-105* single mutant. Moreover, gain-of-function of *MPK3/6* can rescue the inflorescence defects of the *er* mutant. The constitutively activated MKK4/5 and YDA can also at least partially rescue the inflorescence defects of the *er* mutant, generating elongated inflorescences and pedicels when compared with *er-105*. These results support that the YDA-MKK4/5-MPK3/6 cascade functions downstream of the ER signaling pathway to regulate inflorescence architecture in Arabidopsis [84] (Figure 3D).

Regulation of MAPK cascades by ER has been found in other species. The *small grain 1* (*smg1*) mutant with decreased grain size was identified in rice (*Oryza sativa*) through a forward genetic screening, which contains a mutation in *OsMKK4* [188]. A rice MAPK cascade consisting of OsMKKK10, OsMKK4, and OsMPK6 was further identified, which functions to control grain size [189]. A very recent study found that OsERECTA (OsER), a homologous protein of Arabidopsis ER in rice, is involved in controlling the spikelet number per panicle. The spikelet number per panicle is increased and the grain size is reduced in the *oser* mutant. Constitutively activated OsMKKK10 and OsMKK4 can rescue the increased spikelet number phenotype of *oser*, and induce the phosphorylation of OsMPK6 in *oser*. Collectively, OsER acts upstream of the OsMKKK10-OsMKK4-OsMPK6 cascade to control the spikelet number per panicle and grain size [190], which has the potential to be applied in rice agriculture.

## 10. Conclusions and Future Perspectives

MAPK cascades have always been one topic gaining more attention in the research field of signal transduction in plants. Many plant MAPK cascades have already been identified and extensively investigated in the past decades [3,191,192]. However, the upstream signaling components regulating MAPK modules involved in plant growth and development were not effectively identified for a long time. In recent years, RLKs have been revealed to function as key upstream regulators of MAPK cascades to perceive extracellular signals and transduce them to downstream signaling pathways [8,10,53,54,55,93,94,95,113,164,176,177,182].

However, the upstream regulators of some known MAPK cascades involved in plant development have not been identified and characterized yet. For example, the MKK7-MPK6 cascade regulates many developmental processes, such as leaf venation pattern determination and filament elongation [35]. The MKK2-MPK10 cascade is involved in leaf venation architecture [193]. MEKK1 is necessary for external glutamate-mediated root architecture [194]. The ANP3-MKK6-MPK4 cascade plays an important role in male-specific meiotic cytokinesis [15]. As discussed above, the upstream regulators of many MAPK cascades involved in plant development are RLKs. Whether RLKs also perceive external signals and transduce them through these MAPK cascades without known upstream regulators to modulate plant development is worth further investigation. Moreover, the external signals transduced by these MAPK cascades and the so-far unknown RLKs will be important research topics in the near future.

RLKs play pivotal roles in mediating signaling pathways to regulate many important biological processes in plants. For example, some RLKs and small peptides mediate communications between the male and female during double fertilization, a crucial process for generating new generations of plants. FER, a *Catharanthus roseus* receptor-like kinase 1-like (CrRLK1L) RLK, is involved in female-mediated pollen tube reception [195]. ANXUR 1 (ANX1) and ANX2, two other CrRLK1L RLKs, are specifically expressed at the tip of pollen tube to regulate pollen cell wall integrity and pollen tube growth [196,197]. LURE1, a peptide secreted from the synergids, is perceived by POLLEN RECEPTOR LIKE KINASE 6 (PRK6), MALE DISCOVERER 1 (MDIS1), MDIS1-INTERACTING RECEPTOR LIKE KINASE 1 (MIK1) and MIK2 to guide pollen tube growth [198,199,200]. However, the downstream components of these RLK-mediated signaling pathways are still largely unknown. Whether MAPK cascades function downstream of these RLKs to regulate the processes is an intriguing question.

In fact, RLKs perceive extracellular signals and transmit the signals to intracellular transcription factors through a couple of pathways. Except BRI1- and BAK1-mediated brassinosteroid signaling that functions in a MAPK-independent manner [201], most known RLK-mediated signaling pathways recruit MAPK cascades. In plants, the MAPK protein family usually contains dozens of MAPKKK, MAPKK and MPK, implying that many MAPK cascades can be formed. Although other MAPK modules, such as the MKKK3/5-MKK4/5-MPK3/6 cascade regulating plant immunity downstream of PAMP receptors [163], have been identified, the YDA-MKK4/5-MPK3/6 module is likely the most common one that regulates plant development downstream of RLKs. This brings a question: Is YDA-MKK4/5-MPK3/6 a “RLK-specific” signaling cascade? One explanation is that YDA, MKK4/5, and MPK3/6 are the most thoroughly studied MAPKs in Arabidopsis, and the functions of many other MAPKs have not been revealed. For instance, there are 80 MAPKKKs existing in Arabidopsis, but only a few of them, like YDA, MEKK1, and CONSTITUTIVE TRIPLE RESPONSE 1 (CTR1), have been intensively investigated [87,88,202,203,204,205,206,207]. On the other hand, except having functions in RLK-mediated signaling pathways, MPK6 is also extensively involved in phytohormone-triggered signaling pathway [71,182,208]. Along with the deepening of research, it is possible that some novel MAPK modules functioning downstream of RLK-mediated signaling pathways to regulate plant development will be identified.

The same YDA-MKK4/5-MPK3/6 cascade is often recruited by various RLKs to regulate distinct developmental processes, such as embryo development [86], stomatal initiation [164], and inflorescence architecture [84]. Moreover, even the YDA-MKK4/5-MPK3/6 cascade is employed by ER to regulate both stomatal development and inflorescence architecture [84,164]. Therefore, how the signaling specificities of the MAPK cascade in distinct pathways is determined is another intriguing question. One possible explanation is that specific expression patterns of upstream ligands and RLKs can at least partially determine the MAPK cascade specificity, which then transduces the signal to distinct substrates and finally functions in different developmental processes. In addition, after different RLKs recognize the corresponding ligands, the recruited MAPKs may be phosphorylated differentially, resulting in distinct phosphorylation patterns that then possibly determine the specificity of the whole signaling pathway.

RLCKs, a group of RLKs without the extracellular domain, can interact directly with RLKs to regulate plant development and immunity [209]. For example, SSP interacts with ZAR1 to control the zygote asymmetric division [86]; CAST AWAY (CST) can interact with HAE to inhibit organ abscission [210]; RPM1-INDUCED PROTEIN KINASE (RIPK) and FER form a receptor complex that perceives RALF1 to inhibit root growth [211]; LOST IN POLLEN TUBE GUIDANCE 1/2 (LIP1/2) function as components of the receptor complex perceiving the LURE1 signal to mediate micropylar pollen tube guidance [212]; *M*-locus protein kinase (MLPK) transduces the self-incompatibility signal by interacting with the *S*-receptor kinase (SRK) in *Brassica* [213,214]. However, the current findings show that only SSP interacts with both ZAR1 and YDA to regulate the zygote asymmetric division [86], suggesting that RLCKs may bridge RLKs and MAPK cascades to regulate plant development. More RLCKs should be investigated to reveal their possible roles in RLK-MAPK-mediated signaling pathways.
